# Supplementary material for: Effects of Controlled Ozone Exposure on Circulating microRNAs and Vascular and Coagulation Biomarkers: A Mediation Analysis
Source: Noncoding RNA. 2023 Aug 1;9(4):43. doi: 10.3390/ncrna9040043 (PMC10459325; doi:10.3390/ncrna9040043)
Supplement: Supplementary file 1 [file ncrna-09-00043-s001.zip › ncrna-2184418-supplementary.pdf]

Supplemental Table 1. Descriptive statistics of all miRNA parameters.

miRNAs	CTL (n=6)		FO (n=7)		OO (n=10)		All (n=23)
	Filtered air	Ozone	Filtered air	Ozone	Filtered air	Ozone	
let-7d-5p	30.9 (24.8, 37.1)	36.2 (27, 45.5)	32.6 (25.2, 40.1)	32.7 (23.4, 42.0)	29.4 (24.9, 33.9)	28.9 (24.0, 33.8)	31.4 (29.1, 33.6)
let-7e-5p	4.2 (3.6, 4.8)	5.3 (3.5, 7.0)	3.5 (1.8, 5.1)	4.1 (2.8, 5.4)	3.5 (2.3, 4.6)	4.1 (3.2, 4.9)	4.0 (3.6, 4.5)
let-7g-5p	32.9 (26.4, 39.4)	30.8 (25.1, 36.4)	29.1 (21.6, 36.7)	31.9 (26.8, 37.0)	37.3 (26.1, 48.4)	28.7 (22.2, 35.2)	31.9 (29.0, 34.8)
let-7i-5p	58.7 (45.1, 72.3)	58 (43.5, 72.4)	66.1 (50.7, 81.5)	64 (52.5, 75.6)	63.7 (53.5, 73.8)	65.1 (59.1, 71.0)	63.0 (59.3, 66.8)
miR-375	1.0 (0.3, 1.7)	0.9 (0.4, 1.4)	0.9 (0.3, 1.5)	2.1 (0.4, 3.9)	0.7 (0.3, 1.1)	1.0 (0.5, 1.5)	1.1 (0.8, 1.4)
miR-103a-3p	177.2 (150.5, 203.9)	178.2 (143.4, 213)	190.8 (151.9, 229.8)	167.3 (120, 214.7)	180.6 (155.1, 206.0)	167.5 (151.1, 184.0)	176.5 (166.2, 186.8)
miR-106b-5p	50.1 (42.8, 57.3)	52.1 (46.6, 57.6)	51.4 (40.4, 62.4)	50.6 (41.1, 60.0)	55.4 (40.1, 70.6)	49.9 (43.5, 56.3)	51.7 (48.1, 55.4)
miR-122-5p	66.2 (39.4, 93)	127.9 (51.3, 204.5)*	62.2 (22.9, 101.4)	66.8 (44.9, 88.7)	57.8 (46.2, 69.4)	64.2 (32.4, 96.1)	71.5 (58.4, 84.5)
miR-124-3p	0.1 (0.0, 0.1)	0.1 (0.0, 0.2)	0.1 (0.0, 0.3)	0.2 (0.0, 0.4)	0.2 (0.0, 0.4)	0.2 (0.0, 0.5)	0.2 (0.1, 0.2)
miR-125a-5p	0.2 (0.0, 0.4)	0.1 (0.0, 0.3)	0.1 (-0.1, 0.4)	0.3 (0.1, 0.5)	0.2 (-0.1, 0.5)	0.2 (0.1, 0.3)	0.2 (0.1, 0.3)
miR-125b-5p	0.1 (-0.1, 0.3)	0.2 (0.0, 0.3)	0.3 (0.0, 0.6)	0.1 (0.0, 0.1)*	0.1 (0.0, 0.2)	0.2 (0.0, 0.4)	0.2 (0.1, 0.2)
miR-126-3p	78.1 (70.9, 85.4)	79.0 (62.9, 95)	76.0 (65.8, 86.3)	79.8 (57.7, 101.9)	80.6 (70.9, 90.4)	78.6 (70.0, 87.1)	78.8 (74.7, 82.9)
miR-133a-3p	0.8 (-0.2, 1.8)	0.4 (-0.1, 0.9)	0.6 (-0.2, 1.4)	0.9 (-0.4, 2.1)	0.5 (0.0, 1.0)	0.6 (0.2, 1.0)	0.6 (0.4, 0.9)
miR-133b	0.7 (-0.3, 1.7)	0.3 (-0.2, 0.7)	0.5 (-0.2, 1.1)	0.6 (-0.3, 1.6)	0.5 (-0.1, 1.2)	0.4 (0.1, 0.8)	0.5 (0.3, 0.7)
miR-142-3p	1.1 (0.3, 1.8)	1.1 (0.0, 2.2)	0.7 (0.3, 1.1)	1.0 (0.4, 1.6)	0.9 (0.4, 1.4)	0.9 (0.6, 1.3)	0.9 (0.7, 1.1)
miR-144-5p	0.14 (-0.07, 0.35)	0.16 (0.0, 0.32)	0.01 (-0.01, 0.03)	0.19 (0.04, 0.34)*	0.16 (-0.02, 0.34)	0.09 (-0.02, 0.20)	0.12 (0.07, 0.18)
miR-145-5p	4.3 (1.7, 6.9)	3.8 (2.6, 5.1)	3.8 (1.6, 6.0)	3.9 (2.6, 5.3)	4.2 (3.3, 5.1)	4.5 (3.1, 5.8)	4.1 (3.6, 4.6)
miR-146a-5p	99.8 (83.0, 116.5)	99.1 (81.5, 116.7)	129.0 (106.2, 151.8)	119.1 (90.0, 148.2)	112.2 (94.9, 129.6)	110.7 (99.0, 122.5)	112.2 (105.3, 119.1)
miR-150-5p	21.5 (13.1, 30.0)	36.1 (7.7, 64.4)	27.1 (12.7, 41.4)	45.1 (14.6, 75.6)	43.2 (22.2, 64.3)	43.3 (19.5, 67.1)	37.3 (29.3, 45.4)
miR-155-5p	0.4 (-0.2, 1.1)	0.6 (0.3, 1.0)*	0.8 (0.3, 1.3)	0.5 (0.1, 1.0)	0.3 (0.0, 0.7)	0.7 (0.4, 1.0)	0.6 (0.4, 0.7)
miR-15b-5p	84.8 (72.3, 97.2)	90.6 (80.7, 100.5)	82.0 (66.4, 97.5)	84.2 (75.2, 93.1)	81.6 (74.6, 88.7)	84.8 (75.2, 94.4)	84.3 (80.8, 87.8)
miR-16-5p	1060.3 (807.4, 1313.1)	1156.2 (809.5, 1502.9)	1165.5 (857.5, 1473.6)	1223.1 (863.3, 1582.8)	1174.3 (878.7, 1469.9)	1170.6 (937.1, 1404.2)	1162.4 (1063.4, 1261.3)
miR-17-5p	282.5 (265.4, 299.6)	280.8 (255.8, 305.9)	302.4 (268.6, 336.2)	285.0 (274.9, 295.1)	289.2 (279.4, 298.9)	287.8 (274.8, 300.9)	288.3 (282.1, 294.5)
miR-181b-5p	3.9 (2.3, 5.5)	4.3 (3.6, 5.0)	2.3 (1.3, 3.4)	3.7 (2.9, 4.5)	3.6 (1.4, 5.8)	4.0 (2.6, 5.3)	3.6 (3.1, 4.2)

Supplemental Table 1 continued

miR-18a-5p	11.4 (8.5, 14.3)	10.7 (6.3, 15.0)	11.8 (9.5, 14.0)	10.5 (8.9, 12.2)	10.2 (9.2, 11.3)	9.7 (7.3, 12.2)	10.6 (9.8, 11.4)
miR-192-5p	5.1 (3.2, 7.1)	7.4 (3.6, 11.1)	5.8 (2.3, 9.3)	7.8 (4.9, 10.6)	5.4 (3.9, 6.9)	6.1 (3.5, 8.6)	6.2 (5.3, 7.1)
miR-194-5p	2.5 (1.2, 3.9)	3.6 (1.4, 5.9)	2.3 (1.3, 3.3)	3.1 (2.3, 3.8)	2.3 (1.7, 2.8)	2.8 (1.9, 3.7)	2.7 (2.4, 3.1)
miR-195-5p	73 (46.4, 99.6)	87.6 (43.4, 131.8)	57.2 (43, 71.4)	62.7 (46.5, 78.9)	71.7 (49.4, 93.9)	61.3 (46.2, 76.3)	68.1 (60.2, 76)
miR-199a-3p	55.3 (44.5, 66.1)	52.3 (33.6, 70.9)	52.4 (36.5, 68.3)	51.1 (38.1, 64.1)	55.0 (45.9, 64.2)	50.0 (39.1, 60.9)	52.6 (48.5, 56.8)
miR-199a-5p	31.4 (25.8, 37.0)	26.7 (17.5, 36)	33.1 (26.3, 39.9)	27.0 (20.4, 33.5)	28.9 (24.2, 33.6)	26.2 (21.1, 31.3)	28.7 (26.6, 30.8)
miR-19a-3p	15.2 (8.9, 21.5)	11.2 (9.5, 12.8)	12.4 (9, 15.9)	13 (8.7, 17.3)	15.1 (11.5, 18.8)	10.5 (8.3, 12.8)*	12.9 (11.6, 14.2)
miR-1-3p	0.2 (-0.1, 0.6)	0.1 (0.0, 0.3)	0.3 (-0.1, 0.6)	0.3 (-0.1, 0.7)	0.2 (0.0, 0.5)	0.2 (0.0, 0.4)	0.2 (0.1, 0.3)
miR-208a-3p	0.2 (-0.2, 0.5)	0.1 (-0.1, 0.3)	0.2 (-0.1, 0.4)	0.2 (0.0, 0.4)	0.2 (0.0, 0.3)	0.1 (0.0, 0.3)	0.2 (0.1, 0.2)
miR-208b-3p	0.2 (-0.1, 0.4)	0.2 (0.1, 0.3)	0.2 (0.0, 0.4)	0.2 (-0.1, 0.6)	0.2 (0.0, 0.4)	0.2 (0.1, 0.3)	0.2 (0.1, 0.3)
miR-20b-5p	119.3 (100.1, 138.5)	119.9 (92.5, 147.2)	100 (86.3, 113.8)	103.6 (79.6, 127.7)	110.5 (82.7, 138.4)	98.7 (82.3, 115.1)	107.7 (99.8, 115.6)
miR-210-3p	0.9 (0.3, 1.6)	0.6 (0.1, 1.1)	0.8 (0.5, 1.1)	1.3 (0.6, 2.0)	0.7 (0.3, 1.1)	1.0 (0.6, 1.3)	0.9 (0.7, 1.0)
miR-214-3p	1.1 (0.4, 1.9)	1.3 (0.2, 2.4)	0.5 (0.2, 0.9)	0.9 (0.3, 1.4)	1.0 (0.4, 1.6)	0.7 (0.3, 1.0)	0.9 (0.7, 1.1)
miR-21-5p	27.2 (18.2, 36.2)	21.4 (14.5, 28.3)	24.6 (14.2, 35.1)	22.2 (15.7, 28.7)	29.2 (20.8, 37.6)	24.2 (19.7, 28.6)	25.1 (22.4, 27.7)
miR-25-3p	89.8 (74.9, 104.8)	90.4 (63.6, 117.2)	95.6 (67.8, 123.5)	92.6 (72.8, 112.4)	86.9 (68.1, 105.8)	90.7 (70.5, 111.0)	90.8 (83.6, 97.9)
miR-26a-5p	117.5 (97.7, 137.3)	113.9 (87.6, 140.1)	122.3 (96.5, 148.1)	109.1 (81.1, 137.1)	105.1 (87.7, 122.5)	108.6 (92.2, 125.0)	111.8 (104.6, 119.1)
miR-26b-5p	6.6 (4.8, 8.4)	6.1 (5.4, 6.8)	5.3 (4.4, 6.3)	4.3 (2.9, 5.8)	5.2 (3.1, 7.4)	4.6 (3.9, 5.2)	5.3 (4.7, 5.8)
miR-27a-3p	46.9 (41.0, 52.7)	48.7 (36.8, 60.7)	50.0 (43.1, 56.8)	55.5 (47, 64.1)	53.9 (45.1, 62.7)	51.1 (40.9, 61.4)	51.4 (48.2, 54.6)
miR-27b-3p	31.8 (30.0, 33.6)	32.3 (26.7, 37.9)	32.7 (28.1, 37.3)	35 (29.6, 40.5)	34.8 (28.1, 41.5)	32.1 (26.0, 38.2)	33.2 (31.2, 35.2)
miR-28-5p	14.8 (12.4, 17.3)	13.7 (6.1, 21.3)	13.0 (8.6, 17.3)	11.0 (8.2, 13.9)	12.6 (8.6, 16.6)	13.5 (11, 16)	13.0 (11.7, 14.4)
miR-29a-3p	21.7 (16.2, 27.3)	26.9 (19.8, 34)	23.7 (16.9, 30.5)	29.3 (23.3, 35.3)	30.9 (22.2, 39.6)	30.1 (24.0, 36.2)	27.7 (25.1, 30.3)
miR-29b-3p	8.4 (7.0, 9.7)	7.9 (5.4, 10.4)	7.6 (3.7, 11.4)	7.9 (6.7, 9.0)	10.4 (4.9, 15.9)	8.7 (7.4, 10.1)	8.6 (7.4, 9.8)
miR-30a-5p	0.7 (0.4, 1.0)	0.9 (0.3, 1.6)	0.8 (0.5, 1.1)	1.0 (0.6, 1.5)	0.7 (0.3, 1.2)	0.6 (0.3, 0.9)	0.8 (0.7, 0.9)
miR-320b	102.9 (93.9, 112.0)	88.3 (74.4, 102.2)	100.0 (89.7, 110.3)	98.5 (85.9, 111.0)	85.6 (74.1, 97.1)	94.6 (81.2, 108.0)	94.3 (89.8, 98.8)
miR-328-3p	9.6 (6.5, 12.6)	7.4 (2.1, 12.8)	9.3 (6.2, 12.3)	8.1 (4.7, 11.5)	5.7 (3.7, 7.7)	7.2 (4.7, 9.7)	7.7 (6.6, 8.7)
miR-335-5p	5.9 (2.8, 8.9)	5.1 (3.0, 7.1)	4.1 (2.7, 5.6)	4.5 (1.6, 7.5)	5.4 (3.2, 7.6)	5.1 (3.7, 6.5)	5.0 (4.3, 5.7)
miR-337-5p	2.1 (0.6, 3.6)	2.2 (-0.6, 4.9)	1.6 (0.6, 2.6)	1.7 (0.2, 3.2)	1.4 (0.6, 2.2)	2.0 (0.6, 3.4)	1.8 (1.3, 2.3)

Supplemental Table 1 continued

miR-342-3p	11 (9.1, 12.9)	12.7 (9.8, 15.5)	11.9 (8.6, 15.3)	18.1 (11.7, 24.5)*	15.4 (11.6, 19.2)	15.8 (12, 19.7)	14.4 (12.9, 16)
miR-34a-5p	0.5 (0.0, 1.0)	1.6 (-0.3, 3.5)*	1.2 (0.7, 1.6)[#]	1.1 (-0.1, 2.3)	0.8 (0.4, 1.2)	1.1 (0.5, 1.7)	1.0 (0.7, 1.3)
miR-363-3p	2.2 (0.9, 3.5)	2.6 (0.9, 4.4)	1.8 (0.7, 2.9)	1.4 (0.7, 2.1)	1.5 (0.8, 2.2)	1.1 (0.4, 1.7)	1.7 (1.3, 2.0)
miR-370-3p	0.4 (-0.1, 0.8)	0.6 (0.3, 0.9)	0.5 (0.1, 0.9)	0.5 (0.2, 0.9)	0.4 (0.1, 0.8)	0.5 (0.1, 0.8)	0.5 (0.4, 0.6)
miR-423-5p	70.9 (60.8, 81.1)	70.6 (50.8, 90.4)	77.1 (64.2, 89.9)	84.1 (70.5, 97.6)	69.6 (61.7, 77.4)	75.7 (61.9, 89.5)	74.6 (70.1, 79)
miR-433-3p	0.3 (-0.3, 1.0)	0.2 (-0.2, 0.6)	0.6 (0.3, 1.0)	0.6 (-0.2, 1.3)	0.5 (0.0, 1.0)	0.3 (-0.1, 0.7)	0.4 (0.2, 0.6)
miR-451a	800.1 (432, 1168.2)	727.6 (379.9, 1075.4)	639.1 (284.2, 994.1)	757.8 (414.9, 1100.8)	795.8 (540.5, 1051.1)	746.0 (521.3, 970.7)	747.0 (647.7, 846.3)
miR-485-3p	3.6 (0.9, 6.3)	3.0 (1.3, 4.6)	2.0 (0.4, 3.6)	1.6 (0.4, 2.7)	2.5 (0.7, 4.3)	2.7 (1.1, 4.3)	2.5 (1.9, 3.1)
miR-486-5p	90.3 (41.9, 138.7)	83.3 (57.1, 109.4)	81.3 (48.2, 114.4)	99.5 (65.6, 133.5)	95.8 (63.2, 128.4)	92.5 (72.1, 112.9)	91.1 (80.5, 101.7)
miR-499a-5p	0.2 (-0.2, 0.7)	0.3 (0.1, 0.4)	0.3 (-0.1, 0.7)	0.1 (0.0, 0.3)	0.1 (0.0, 0.2)	0.1 (0.0, 0.1)	0.2 (0.1, 0.2)
miR-505-5p	0.6 (0.1, 1.1)	0.4 (0.0, 0.7)	0.3 (-0.1, 0.7)	0.6 (0.2, 1.0)	0.3 (0.0, 0.6)	0.4 (0.1, 0.7)	0.4 (0.3, 0.5)
miR-590-5p	0.7 (0.3, 1.2)	0.3 (0.1, 0.5)	0.6 (0.3, 0.9)	0.6 (0.1, 1.0)	0.7 (0.3, 1.1)	0.8 (0.3, 1.2)	0.6 (0.5, 0.8)
miR-92a-3p	111.1 (84.0, 138.1)	103.2 (84.4, 122.1)	112.4 (96.2, 128.5)	124.6 (103.0, 146.1)	110.8 (88.9, 132.7)	100.4 (86.8, 113.9)	109.9 (103, 116.8)
miR-93-5p	249.6 (226.5, 272.7)	235.0 (209.1, 261)	245.7 (221.1, 270.3)	249.2 (223.6, 274.9)	253.0 (234.8, 271.2)	246.4 (225.9, 266.9)	247.1 (239.6, 254.6)

For descriptive statistics, mean and 95% confidence intervals of all 65 miRNAs post filtered air and O_3 exposure were presented for each dietary group.
For statistical analysis, data were first log-transformed and a two-factor (O_3 exposure and supplementation status) mixed effects model with a participant-specific random intercept. Pair-wise comparisons were adjusted using Tukey's tests. * $p < 0.05$ indicate significant differences between filtered air and O_3 exposure in each dietary group. [#] $p < 0.05$ indicate significant differences in FO or OO group compared with CTL group on the filtered air or O_3 exposure day.
CTL: control, FO: fish oil, OO: olive oil.

Supplemental Table 2. Parameters of type III statistics of changes in circulating miRNAs affected by experimental conditions.

miRNAs	Ozone exposure		Dietary supplementation		Interaction	
	F value	*p*	*F* value	*p*	*F* value	*p*
let-7d-5p	0.460	0.506	0.910	0.420	0.740	0.488
let-7e-5p	4.210	0.0536	1.500	0.246	0.040	0.961
let-7g-5p	0.430	0.520	0.120	0.891	1.410	0.267
let-7i-5p	0.000	0.997	0.910	0.419	0.100	0.901
miR-375	2.930	0.103	1.580	0.231	0.240	0.790
miR-103a-3p	2.050	0.168	0.040	0.963	0.660	0.527
miR-106b-5p	0.050	0.830	0.010	0.987	0.360	0.702
miR-122-5p	7.090	0.015	1.210	0.320	2.860	0.081
miR-124-3p	0.690	0.421	0.970	0.404	0.080	0.922
miR-125a-5p	1.420	0.256	0.370	0.700	0.270	0.772
miR-125b-5p	0.320	0.588	0.000	0.998	3.090	0.095
miR-126-3p	0.010	0.935	0.120	0.885	0.050	0.954
miR-133a-3p	0.000	0.949	0.500	0.616	0.490	0.625
miR-133b	0.020	0.892	0.070	0.937	0.080	0.920
miR-142-3p	0.300	0.589	0.060	0.942	0.350	0.709
miR-144-5p	6.390	0.0448	1.220	0.359	4.420	0.066
miR-145-5p	0.110	0.746	0.520	0.600	0.220	0.808
miR-146a-5p	0.370	0.549	3.860	0.0383	0.240	0.785
miR-150-5p	3.040	0.0964	0.750	0.487	1.460	0.256
miR-155-5p	2.840	0.116	1.220	0.327	3.050	0.082
miR-15b-5p	1.430	0.245	0.560	0.581	0.070	0.929
miR-16-5p	0.450	0.508	0.100	0.902	0.050	0.955
miR-17-5p	1.080	0.310	1.060	0.365	0.550	0.583
miR-181b-5p	2.630	0.121	0.720	0.500	0.640	0.538
miR-18a-5p	2.230	0.151	0.740	0.489	0.020	0.983
miR-192-5p	4.370	0.0496	0.300	0.746	0.920	0.414
miR-194-5p	7.830	0.0111	0.050	0.955	0.440	0.651
miR-195-5p	0.160	0.696	0.870	0.432	1.130	0.342
miR-199a-3p	0.710	0.410	0.100	0.904	0.220	0.808
miR-199a-5p	7.140	0.0146	0.360	0.700	0.280	0.755
miR-19a-3p	5.470	0.0299	0.040	0.958	2.200	0.137
miR-1-3p	1.070	0.335	0.040	0.957	0.640	0.557
miR-208a-3p	0.040	0.840	0.220	0.809	0.320	0.737
miR-208b-3p	1.540	0.239	0.140	0.867	0.140	0.875
miR-20b-5p	0.110	0.741	1.680	0.212	0.190	0.826
miR-210-3p	0.920	0.351	0.770	0.477	0.750	0.488
miR-214-3p	0.370	0.552	2.310	0.128	2.130	0.148
miR-21-5p	2.810	0.109	0.890	0.427	0.240	0.789

Supplemental Table 2 continued

miR-25-3p	0.000	0.982	0.200	0.817	0.090	0.917
miR-26a-5p	0.720	0.406	0.410	0.672	0.950	0.404
miR-26b-5p	1.480	0.238	3.940	0.0361	0.590	0.563
miR-27a-3p	0.140	0.712	0.520	0.605	0.980	0.392
miR-27b-3p	0.000	0.993	0.120	0.886	0.760	0.479
miR-28-5p	0.200	0.656	0.550	0.584	0.660	0.526
miR-29a-3p	2.830	0.108	1.530	0.240	0.800	0.461
miR-29b-3p	0.000	0.960	0.930	0.409	0.450	0.643
miR-30a-5p	0.020	0.876	1.300	0.295	0.140	0.869
miR-320b	0.360	0.553	1.640	0.218	3.110	0.067
miR-328-3p	0.160	0.690	1.130	0.344	1.420	0.265
miR-335-5p	0.050	0.826	0.910	0.419	0.070	0.936
miR-337-5p	0.110	0.740	0.450	0.647	0.890	0.428
miR-342-3p	4.080	0.0571	2.110	0.148	1.560	0.234
miR-34a-5p	4.770	0.0441	0.960	0.404	2.950	0.081
miR-363-3p	0.570	0.458	3.210	0.0617	0.440	0.650
miR-370-3p	3.010	0.102	0.040	0.963	0.290	0.752
miR-423-5p	0.480	0.496	1.770	0.196	0.290	0.753
miR-433-3p	0.000	0.986	0.580	0.587	1.790	0.236
miR-451a	0.030	0.857	0.150	0.864	0.690	0.513
miR-485-3p	0.230	0.638	1.270	0.307	1.580	0.234
miR-486-5p	0.630	0.436	0.080	0.923	0.500	0.616
miR-499a-5p	0.100	0.758	0.030	0.974	1.320	0.315
miR-505-5p	0.240	0.632	0.060	0.938	2.550	0.123
miR-590-5p	1.220	0.284	0.880	0.431	0.600	0.561
miR-92a-3p	0.090	0.770	1.160	0.335	1.170	0.331
miR-93-5p	1.100	0.306	0.200	0.823	0.740	0.491

For statistical analysis, data were first log-transformed and a two-factor (O_3 exposure and supplementation status) mixed effects model with a participant-specific random intercept. *F* and *p* values of the type III statistics of the mixed-effects model are presented for each miRNA.
CTL: control, FO: fish oil, OO: olive oil.

Supplemental Table 3. Descriptive statistics of blood protein parameters

Biomarkers	CTL (n=6)		FO (n=7)		OO (n=10)		All (n=23)
	Filtered air	Ozone	Filtered air	Ozone	Filtered air	Ozone	
CRP	525 (88, 962)	546.7 (3, 1090.3)	788.8 (-45.7, 1623.3)	792.2 (-124.4, 1708.8)	794.6 (-203.1, 1792.4)	1347.6 (-888.9, 3584.1)	846.1 (356.7, 1335.5)
D-Dimer	130.5 (82.5, 178.5)	121.1 (74.1, 168)	170.7 (63.3, 278.1)	151.1 (59.5, 242.7)	132.2 (86, 178.5)	125.2 (83.1, 167.3)	137.8 (116, 159.5)
E-selectin	44 (28.1, 59.9)	40.9 (23.1, 58.6)	48.4 (31.8, 65.1)	43.5 (28.5, 58.4)*	43.2 (33.7, 52.7)	42.4 (32.3, 52.4)	43.7 (39.3, 48)
IL-6	0.5 (0.4, 0.6)	1.1 (0.8, 1.5)*	0.8 (0.3, 1.3)	1.1 (0.6, 1.5)	0.6 (0.3, 1)	1.4 (0.9, 1.8)*	0.9 (0.8, 1.1)
IL-8	2.2 (1.5, 2.8)	2.4 (1.8, 3)	2.5 (1.8, 3.2)	2.8 (1.6, 3.9)	2.5 (2, 3)	2.4 (2, 2.8)	2.5 (2.2, 2.7)
IL-1β	0.1 (0.1, 0.1)	0.1 (0.1, 0.1)	0.1 (0.1, 0.1)#	0.1 (0.1, 0.1)	0.1 (0.1, 0.1)#	0.1 (0.1, 0.1)	0.1 (0.1, 0.1)
SAA	1426.8 (346.9, 2506.8)	1073.2 (319.3, 1827.1)	948.2 (378.4, 1518)	828.3 (331.2, 1325.4)	2526.6 (-1557.9, 6611.1)	5295.7 (-5006.6, 15598.1)	2296.9 (172.1, 4421.7)
TNFα	0.8 (0.6, 1)	0.8 (0.6, 1)	0.7 (0.5, 0.9)	0.7 (0.5, 0.8)	0.9 (0.7, 1)	0.8 (0.7, 1)	0.8 (0.7, 0.8)
sICAM1	202 (172.4, 231.5)	202.7 (174, 231.3)	212.8 (171.4, 254.2)	213.9 (173, 254.9)	215 (194.7, 235.3)	213.4 (190.4, 236.4)	210.9 (201, 220.7)
sVCAM1	251.3 (215.9, 286.8)	256.1 (214.5, 297.6)	233.7 (188.7, 278.7)	240.5 (181.9, 299.1)	291.2 (248.4, 333.9)	294.9 (253.1, 336.8)	265.8 (249, 282.5)
vWF	11.8 (8.9, 14.7)	13.6 (9.1, 18)	10.4 (5.8, 14.9)	10 (6.5, 13.6)	12.5 (9.7, 15.3)	11.6 (9.3, 13.9)	11.7 (10.5, 12.8)

For descriptive statistics, mean and 95% confidence intervals of all 11 protein biomarkers post filtered air and O_3 exposure were presented for each dietary group.
For statistical analysis, data were first log-transformed and a two-factor (O_3 exposure and supplementation status) mixed effects model with a participant-specific random intercept. Pair-wise comparisons were adjusted using Tukey's tests. * $p < 0.05$ indicate significant differences between filtered air and O_3 exposure in each dietary group. # $p < 0.05$ indicate significant differences in FO or OO group compared with CTL group on the filtered air or O_3 exposure day.
CRP: c-reactive protein, CTL: control, IL: interleukin, FO: fish oil, OO: olive oil, SAA: serum amyloid A, sICAM1: soluable intercellular adhesion molecule 1, sVCAM1: soluble vascular cell adhesion molecule 1, TNFα: tumor necrosis factor alpha, vWF: von Willebrand factor.
